# Biogenic Synthesis, Characterization, and In Vitro Biological Evaluation of Silver Nanoparticles Using *Cleome brachycarpa*

**DOI:** 10.3390/plants12071578

**Published:** 2023-04-06

**Authors:** Ayesha Ahmed Sumra, Maryam Zain, Tahira Saleem, Ghulam Yasin, Muhammad Farooq Azhar, Qamar Uz Zaman, Vishwanie Budhram-Mahadeo, Hayssam M. Ali

**Affiliations:** 1 Department of Chemistry, The Women University Multan, Multan 60000, Pakistan; ayeshaahmed@wum.edu.pk (A.A.S.); tslm2011@gmail.com (T.S.); 2 Department of Biochemistry and Biotechnology, The Women University Multan, Multan 60000, Pakistan; maryam.9002@wum.edu.pk; 3 Department of Forestry and Range Management, Bahauddin Zakriya University Multan, Multan 60000, Pakistan; farooqazhar@bzu.edu.pk; 4 Department of Environmental Sciences, The University of Lahore, Lahore 54590, Pakistan; qamar.zaman1@envs.uol.edu.pk; 5 Molecular Biology Development and Disease, University College London, London WC1E 6BT, UK; v.budhram-mahadeo@ucl.ac.uk; 6 Department of Botany and Microbiology, College of Science, King Saud University, Riyadh 11451, Saudi Arabia; hayhassan@ksu.edu.sa

**Keywords:** *Cleome brachycarpa*, silver nanoparticles, antioxidant activity, antidiabetic activity, cytotoxic potential, MCF-7

## Abstract

The therapeutical attributes of silver nanoparticles (Ag-NPs) in both conditions (in vitro and in vivo) have been investigated using different plants. This study focused on the green chemistry approach that was employed to optimize the synthesis of silver nanoparticles (AgNPs) using *Cleome brachycarpa* aqueous extract as a reducing and stabilizing agent. The characterization of obtained *CB*-AgNPs was undertaken using UV-visible spectroscopy, Atomic-force microscopy (AFM), Fourier-Transform Infrared Spectroscopy (FTIR), scanning electron microscopy (SEM), and Energy-Dispersive X-ray (EDX) analysis. Results suggest that *CB*-AgNPs synthesized via stirring produced small-sized particles with more even distribution. The synthesized silver nanoparticles were spherical with a 20 to 80 nm size range. In vitro studies were used to analyze antioxidant, antidiabetic, and cytotoxic potential under different conditions. The results also indicated that *CB*-AgNPs may have significant potential as an antidiabetic in low concentrations, but also exhibited potential antioxidant activity at different concentrations. Moreover, the anticancer activity against the breast cell line (MCF-7) with IC_50_ reached up to 18 μg/mL. These results suggest that green synthesized silver nanoparticles provide a promising phytomedicine for the management of diabetes and cancer therapeutics.

## 1. Introduction

Nanoparticles are important nanotechnology field products that are used in numerous sizes and shapes as an eco-friendly approach [1,2]. Green nanotechnology is a promising new approach for the efficient synthesis of nanoparticles, including gold, zinc, and silver metals [3]. Most importantly, the conformation and distribution of nanoparticles in solution, along with their enhanced surface areas, help stimulate the physio-chemical properties that are significantly advantageous for anti-microbial growth [4], bio-molecular detection and diagnostics [5], cosmetics [6], biomedical research [7], drug delivery [8], gene delivery [9], and the environment [10]. A diverse range of its applications and advancing capacity to manipulate functions and forms of nanoparticles have increased interest in synthesizing nanoparticles using eco-friendly and effective synthetic techniques.

More recently, the beneficial uses of metal nanoparticles, particularly silver-generated and silver nanoparticles(AgNPs), have proved to carry promising biomedical application potential [11] owing to their antioxidant, antimicrobial, anticancer, and antiplatelet activities and potential angiogenic uses [12,13,14]. However, AgNPs also have numerous biological applications in pharmacology; phytoremediation; photo-electrochemistry; phytomining; single-electron transistors, sensors, and tracers; and wastewater treatment [15,16]. Urban effluent treatment has attracted great attention in developing unique methods including different biological routes to produce nanoparticles of different shapes and sizes. This is important because the physico-chemical methods commonly used to produce nanostructures require a variety of chemicals that are damaging to ecosystem functioning and health [17]. To overcome the challenges of chemical synthesis of AgNPs for biological usage, the present study aimed at testing whether the *Cleome* species could be used to effectively synthesize and characterize AgNPs.

Previous evidence has shown that plant-based generation of AgNPs (green synthesis nanoparticles) can be accomplished using various plant species and different parts of plants, including leaves, stems, roots, buds, fruit, latex, and bark [18]. By employing spectroscopic analysis, it has been demonstrated that the applications of the most influential phytochemicals involved in reducing silver included flavonoids, phenolic acid, phenols, alkaloids, terpenoids, aldehydes, amides, and ketones [19,20]. Quinone and flavones are among the water-soluble phytochemicals that are considered liable for a speedy reduction. Additionally, reducing the aptitude of secondary metabolites from plant extracts has a higher potential ability to synthesize nanoparticles of small sizes with large surface areas [21]. Using plant extracts seems advantageous in AgNP synthesis due to their function as reducing agents (for instance, Ag^+^ to Ag^0^), stabilizing mediators, and avoiding nanoparticle clustering. Therefore, the current study focused on synthesizing AgNPs using *Cleome brachycarpa* extract, and tested the pharmacological properties of nanoparticles.

*Cleome brachycarpa* (punwar) is a ethanomedicinal plant of Pakistan [22] found in the semi-arid to subtropical regions of Punjab, Sindh, KPK and adjoining areas [23,24]. It is commonly named wild mustard and tick-weed, and is widely used in conventional medicines, but is also used in cooking (leaves and fresh branches) [25]. Its seed pods are used to make pickles and local delicacies [26]. According to the Ayurvedic medical system, the extracts of *Cleome brachycarpa* have been used for the treatment of various diseases such as diarrhea, malaria, illness, hypertension, and snakebite. The compounds visconoside, astragalin, kaempferol, quercetin, and kaempferitrin extracted from *C. brachycarpa* leaves have been shown to display hepatoprotective activity against CCl_4_-induced hepatotoxicity [27]. Citrus viscosa 3-O-(200-acetyl)-glucoside, also extracted from this plant, is considered to possess antibacterial and anti-inflammatory properties [25].

Previous studies have also used fruit and leaf extract from other plants (*Calligonum comosum, Azadirachta indica*, and *Caesalpinia pulcherrima*) to synthesize AgNPs that were tested for antibacterial and cytotoxic effects on human breast cancer cells [28]. However, the investigation of antioxidant properties of plant extracts reported in this study [29] and AgNPs synthesized from *C. brachycarpa* are unique and have not been reported before. Therefore, in addition to focusing on the synthesis of AgNPs using *C. brachycarpa* (plant aqueous extract), which functioned as a stabilizer and reducing agent, this study also performed a qualitative pharmacological screening of synthesized AgNPs. Considering the significance of AgNPs and their potential applications in biomedicine, we focused on using the bioreduction method to produce sustainable AgNPs from the aqueous extract of *C. brachycarpa* leaves. The synthesized AgNPs were characterized using UV–visible spectroscopy for surface plasmon resonance bands, FTIR for functional groups responsible for reduction, and SEM with EDX (Energy-Dispersive X-ray) for a determination of the size of synthesized materials. Furthermore, AgNPs obtained from this process were assessed for antioxidants, antidiabetic action, and in vitro anti-cancer potential. We expected to develop a promising method for the green synthesis of AgNPs using *C. brachycarpa* extract, and its potential applications were evaluated.

## 2. Materials and Methods

### 2.1. Collection of Plant Samples

**Scheme 1:**
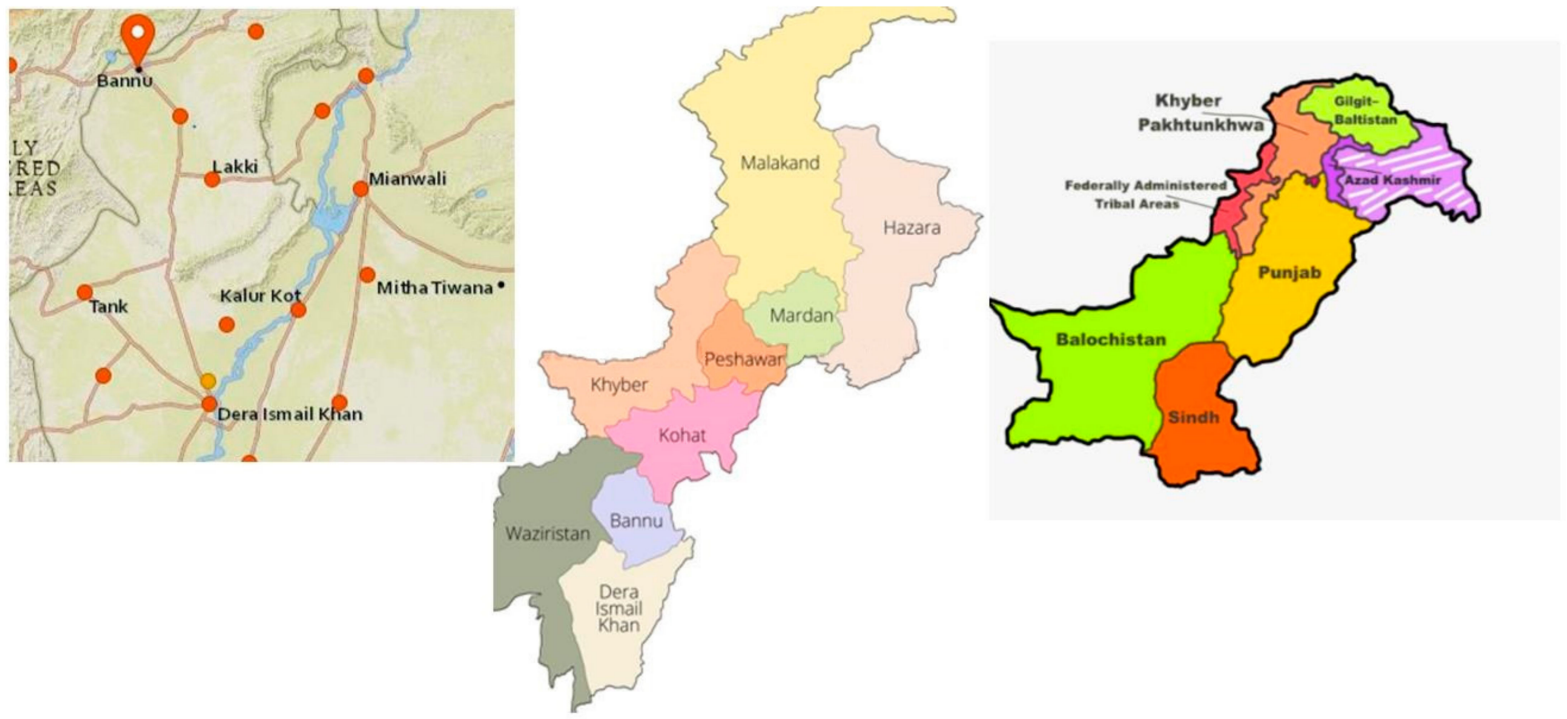
Study area map and geographical distribution of plants in Pakistan.

Collection of the *C. brachycarpa* plant was carried out in March from the KPK province (River Kurram and Hill torrents). The sampled area is located between 32.43 and 33.06 N and between 70.22 and 70.57 E , as shown in the location map (Scheme 1). Plant species were identified and verified based on their vegetative and floral characteristics by Botanist Prof. Dr. Sultan Mehmood from the University of Science and Technology, Bannu (Identification certificate attached in Supplementary Data).

### 2.2. Aqueous Extraction of C. brachycarpa

The *C. brachycarpa* extracts were prepared following the procedure reported by Benakashani et al. [30] with some modifications. After washing, plants were dried at room temperature, then ground into powder form and sieved to remove debris and particles. For the extract preparation, about 3 g of powder was macerated at 30 °C, centrifuged at 150 rpm, and then subjected to sonication (model-Elmasonic, E60H) at 40 °C to homogenize the mixture for 2 h in 35 mL distilled water. The brown filtered solution thus obtained was evaporated and lyophilized to acquire pure extract, which was then stored at 4 °C to synthesize nanoparticles.

### 2.3. Qualitative Phytochemical Study

The extract’s preliminary phytochemical screening was conducted to identify its active constituents, using standard chemical methods reported in the literature [29] and the WHO (World Health Organization) guidelines [31]. A 1% stock solution was prepared for phytochemical screening. The aqueous leaf extract of *C. brachycarpa* was tested conventionally for its phytoconstituents such as steroids, alkaloids, flavonoids, phenolics, glycosides, tannins, saponins, anthracyanins, terpenoids, etc. Detailed conventional qualitative tests are explained in Table 1.

### 2.4. Silver Nanoparticles SYNTHESIS

**Scheme 2:**
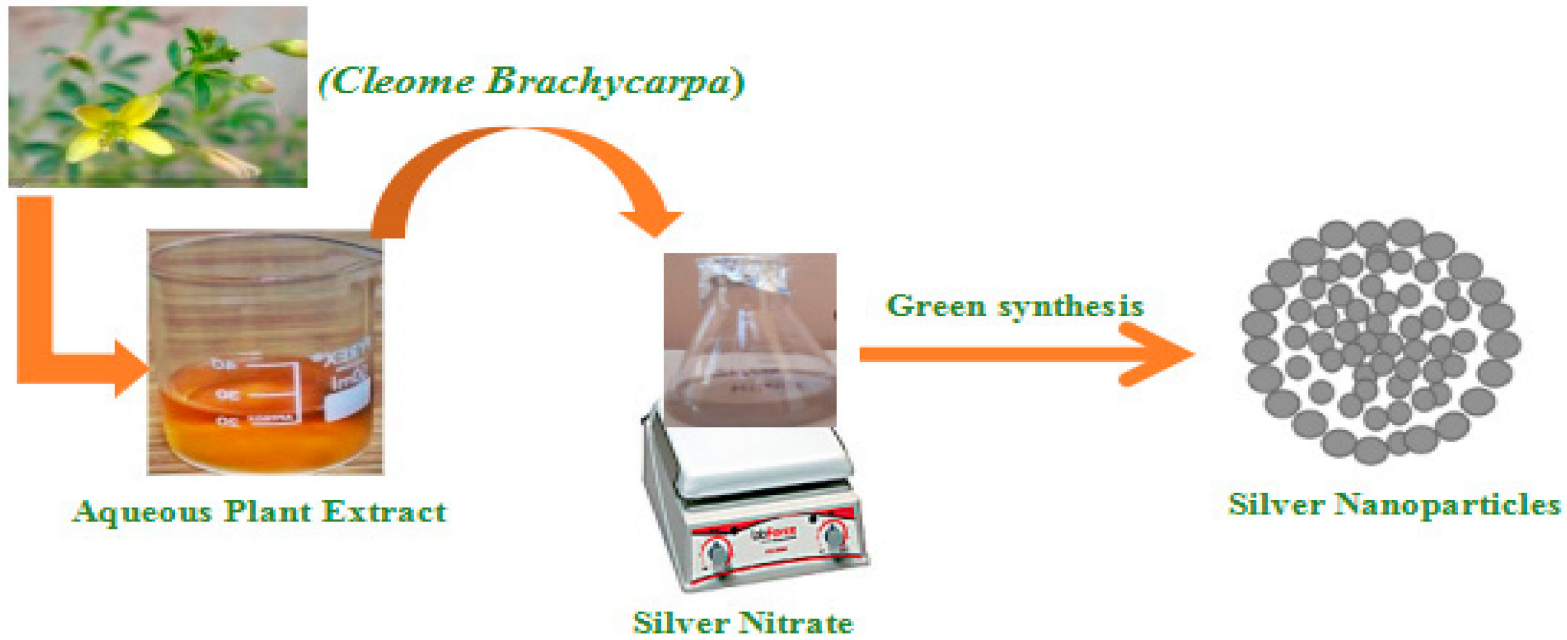
Schematic diagram for biological synthesis of AgNPs.

The procedures described in the literature on biogenic synthesis were followed in the preparation of silver nanoparticles [32]. Aqueous *C. brachycarpa* extract (10 mL) was mixed thoroughly with AgNO_3_ (90 mL, 1 mM) aqueous solution, and the incubation of the mixture was performed at room temperature under static conditions (Scheme 2). The AgNP formation was observed through the color transition from pale yellow to dark brown. The obtained silver nanoparticles were centrifuged at 11,000 rpm for 5 min. The prepared final pellet was dried in an oven at 60 °C as depicted in Scheme 2.

### 2.5. Characterization Techniques

The biosynthesized AgNPs were morphologically, chemically, and physically characterized by complying with standard procedures provided in the literature using diverse analytic techniques such as UV-visible spectroscopy, Fourier-Transform Infrared Spectroscopy (FTIR), scanning electron microscopy (SEM), Energy-Dispersive X-ray (EDX) analysis, and Atomic Force Spectroscopy (AFM) analysis [33].

#### 2.5.1. UV–Visible Spectral Analysis

The formation of AgNPs and their stability was examined at different time intervals by measuring absorbance using a double-beam spectrophotometer (HALO, DB-20, COM 1:6,822,0121) for 24 h at a resolution of 2 nm between 200 nm and 700 nm. The solution color was also noted at different intervals.

#### 2.5.2. FTIR Analysis

The different spectra were compared with each other in order to analyze the fingerprints of *C. brachycarpa* represented by the band located at a wavelength range of 400–4000 cm^−1^ using an FTIR spectrophotometer (Bruker alpha). A total of 2 μg of each mineralized sample was used to form a semitransparent pellet, which allowed light to be transmitted to the detector.

#### 2.5.3. SEM and EDX Analysis

The AgNPs were characterized according to their morphology and size via scanning electron microscopy (SEM) and EDX analysis using a VEGA TESCAN-XMU SEM machine. The AgNPs were evenly spread with carbon tape and sputter-coated with platinum on sample holders. After obtaining SEM images, EDX analysis was performed to confirm the presence of the different elemental compositions of the nanoparticles in the samples, following the method described earlier [34].

#### 2.5.4. Atomic Force Microscope

The AFM was performed using AFM-Agilent 7500 AFM/SPM 3D in the tapping mode. Maximum scan areas were 90 μm × 90 μm. The sample with a cantilever was located by means of a charge-coupled device monitor. Sample imaging related to size was conducted at 10 μm, 5 μm, 2 μm, and 1.2 μm scan widths. The acquired phase and height images thus obtained were analyzed using Asylum Research IGOR PRO-based software.

### 2.6. Antioxidant Activity

#### 2.6.1. DPPH Scavenging Activity

About 1 mL of different concentrations (25–200 μg/mL) of the compounds were added to methanol to prepare 4 mL of DPPH (0.004%) solution. After incubation for 30 min at room temperature, absorbance at 517 nm against a blank was recorded. The percent inhibition (I (%)) of free radicals by DPPH was calculated as follows [35]:

I (%) = ((A_blank_ − A_sample_)/A_blank_) × 100.

#### 2.6.2. Reducing Power Assay

The reducing capacities of nanoparticles were assessed with a ferric-reducing antioxidant power assay. About 2.5 mL of phosphate buffer and 2.5 mL potassium ferricyanide (1% *w*/*v*) were added to different dilutions of plant extracts. The mixture was incubated at 50 °C for 25 min. The mixture was allowed to cool down, and 2.5 mL of 10% trichloroacetic acid solution was added to it to stop the reaction. The upper layer of the solution (2.5 mL) was equally blended with water and a ferric chloride solution of 0.5 mL. After 30 min, absorbance was measured at 700 nm. The control of BHT of various concentrations (25, 50, 75, 100, and 200 μg/mL) was also prepared without adding the sample.

### 2.7. In Vitro Antidiabetic Activity

3,5 Dinitrosalicylic Acid Assay (DNSA)

To measure relative enzyme activity, we used a dinitrosalicylic acid assay with some modifications to previous researchers’ methods [36]. The mixture consisted of 500 μL of sodium phosphate buffer (0.02 M with pH 6.9 and 6 mM sodium chloride), salivary amylase (1 mL), and extracts of 400 μL at a concentration between 0.3 and 1.5 mg/mL^−1^ (*w*/*v*) that was incubated for 10 min at 37 °C. After pre-incubation, in the above buffer, 580 μL of 1% (*w*/*v*) starch solution was added to each tube. Then, the reaction mixture of 1 mL DNSA reagent was added and placed in a boiling water bath for 5 min, cooled at room temperature, and diluted, and absorbance was measured at 540 nm. A value of 100% enzyme activity was represented by control and plant extract was absent. To remove the absorbance induced by plant extract, the controlled extract was added to the reaction mixture, except for the enzyme. To calculate the percent inhibition of alpha-amylase, the following method was used:

% Relative enzyme activity = (enzyme activity test/enzyme activity control) × 100
% inhibition in the alpha - amylase activity = (100 - % Relative enzyme activity)

### 2.8. In Vitro Anti-Cancer Potential

#### 2.8.1. Cell Culture

MCF-7 cells were maintained in Dulbecco’s Modified Eagle’s Medium (DMEM) containing 10% fetal bovine serum and 1% penicillin-streptomycin, which was incubated at 37 °C in a humidified incubator. At 80% confluence, cells were reseeded and passaged twice weekly with Trypsin-EDTA [37].

#### 2.8.2. Cell Viability Assay

To assess the cytotoxic impact of AgNPs, an MTT (3-(4, 5-dimethylthiazol-2-yl)-2, 5-diphenyltetrazolium bromide) assay was conducted using 96-well plates [38,39]. Briefly, MCF-7 cells were trypsinized and transferred into 12-well plates at a density of 1 × 10^5^ cells/well in 1 mL of DMEM. After incubation overnight, cells were exposed to different doses (0–100 μg/mL) of the tested AgNPs and incubated again for 24 h. Before undertaking the MTT assay (i.e., addition of 5% MTT (50 μL), samples were added into each well followed by incubation of cells at 37 °C for 4 h. The media were replaced with 100 μL of DMSO for 15 min and, after that, the optical density (OD) at 570 nm was measured using a microplate reader. The percentage of cytotoxicity was measured with the following equation [40]:

% Cell Viability = (OD of test/OD of control) × 100 % Cytotoxicity = 100 - % cell viability

Statistical Analysis:

All the finding results are presented as mean ± SDs. Experiments were conducted in triplicates. One-way ANOVA was used to determine the significances between group subsets, and *p*-values < 0.05 were considered significant.

## 3. Results

**Figure 1:**
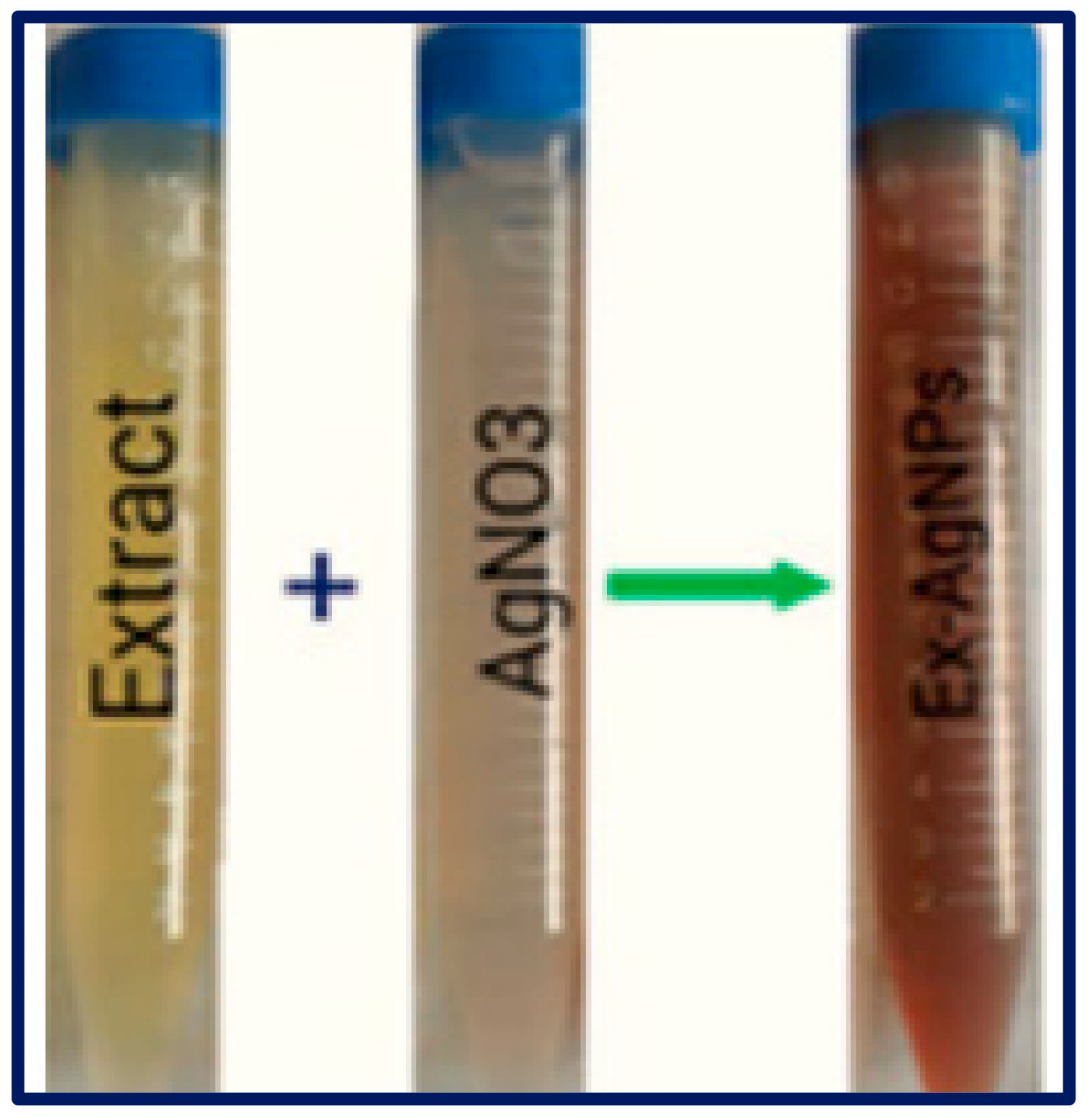
Color change indication of formation of AgNPs.

Various color tests carried out for initial phytochemical analysis of the extract of *C. brachycarpa* revealed the presence of bioactive contents such as phenolics, flavonoids, alkaloids, Tannins, sapnins, glycosides, terpenoids, and steroids. The concentration of glycosides was not detected in the aqueous extract of *C. brachycarpa*, as shown in Table 2. In the present study, the biogenic synthesis of silver nanoparticles was performed without adding any external reducing agents where biomolecules act as capping and stabilizing agents. Different techniques characterize these nanoparticles. The basic indication for the formation of AgNPs is a change in color from yellow to dark brown, as shown in Figure 1.

### 3.1. UV–Visible Spectroscopy

**Figure 2:**
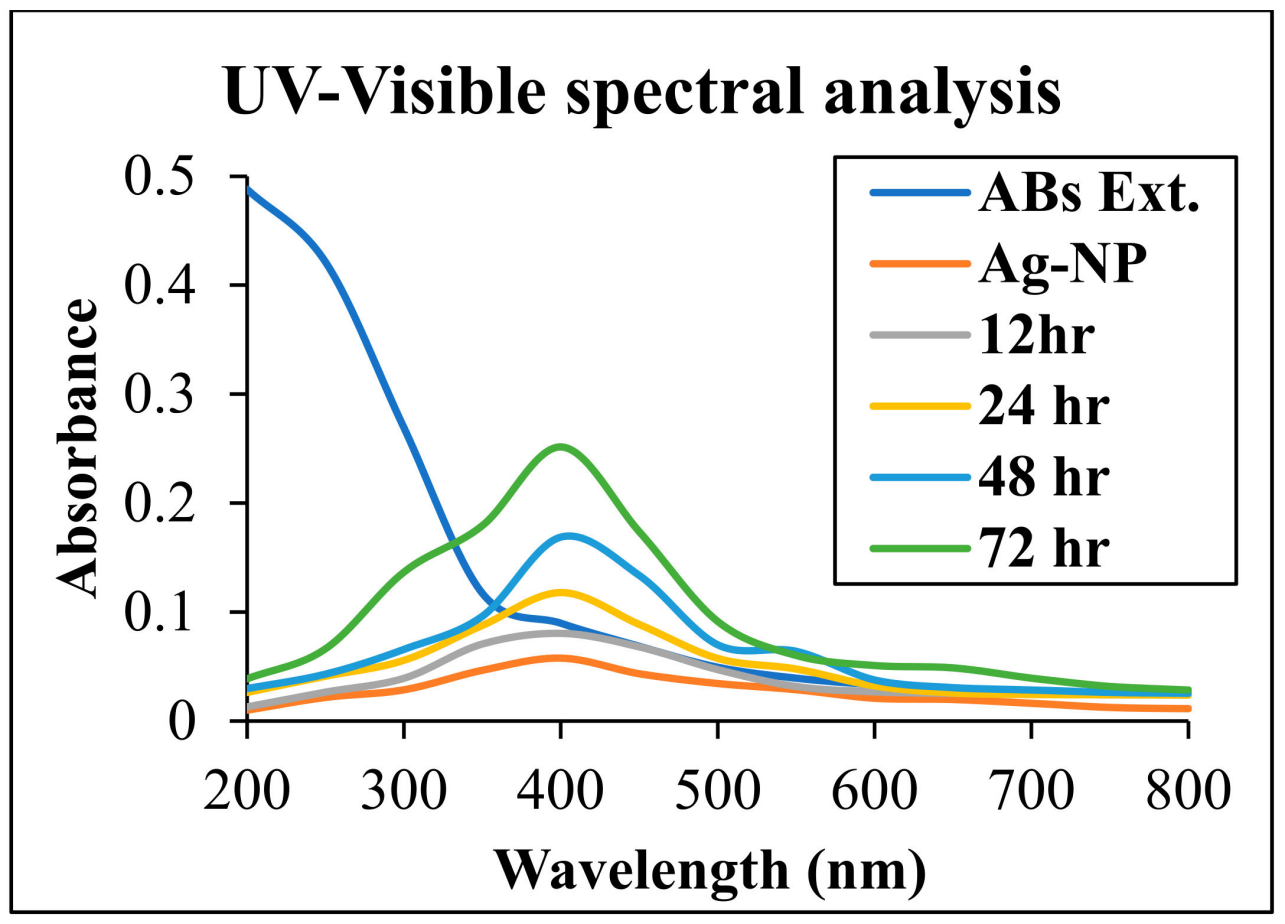
UV–Visible spectrum at different time durations.

**Figure 3:**
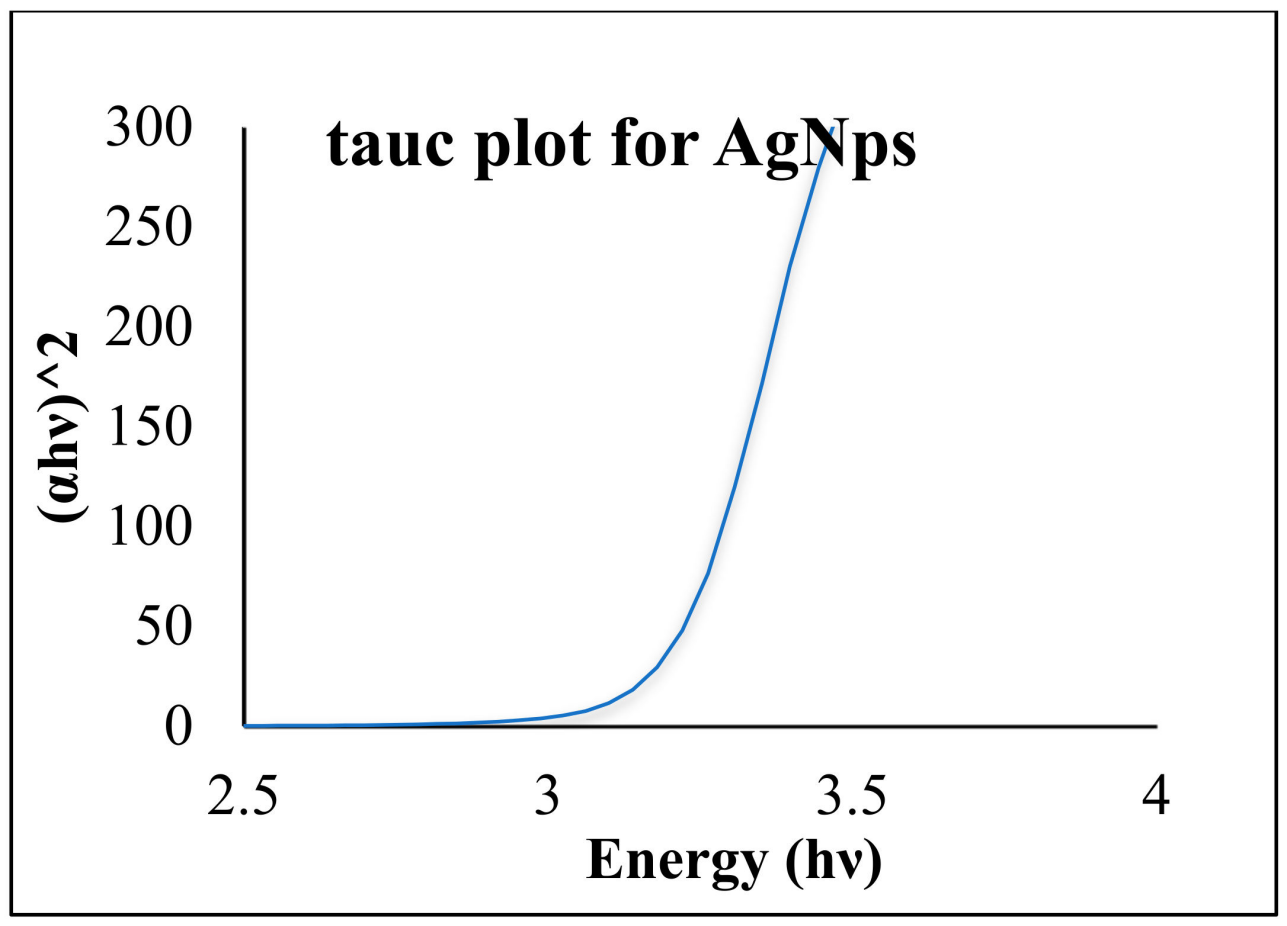
Energy Bandgap determined using Tauc plots.

UV–Visible spectroscopy of extracts of the plant was conducted for bio-reduction of Ag+ ions at a temperature of 40 °C. The extract showed maximum absorbance bands at 285 nm and 375 nm. Band I at 375 nm was assigned for the cinnamoyl system of conjugated aromatics, and the band II at 285 nm indicated phenolics (benzoyl system), as shown in Figure 2. A variation in color was also associated with precise peaks characterized by maxima centered at 285 nm and 375 nm. The synthesized silver nanoparticles were interpreted by scanning the UV–visible spectra. The spectrum showed a significant difference in maxima. The highest absorption peak was recorded at 437 nm, while the lowest peak was recorded at 190 nm. The formulated metal nanoparticles were well-established due to the polyphenolic compounds in the extract that re-stained the accumulation. A significant energy band gap of 3.2 eV was calculated by using Tauc plots shown in Figure 3.

### 3.2. Fourier-Transform Infrared Spectroscopy (FTIR) Analysis

**Figure 4:**
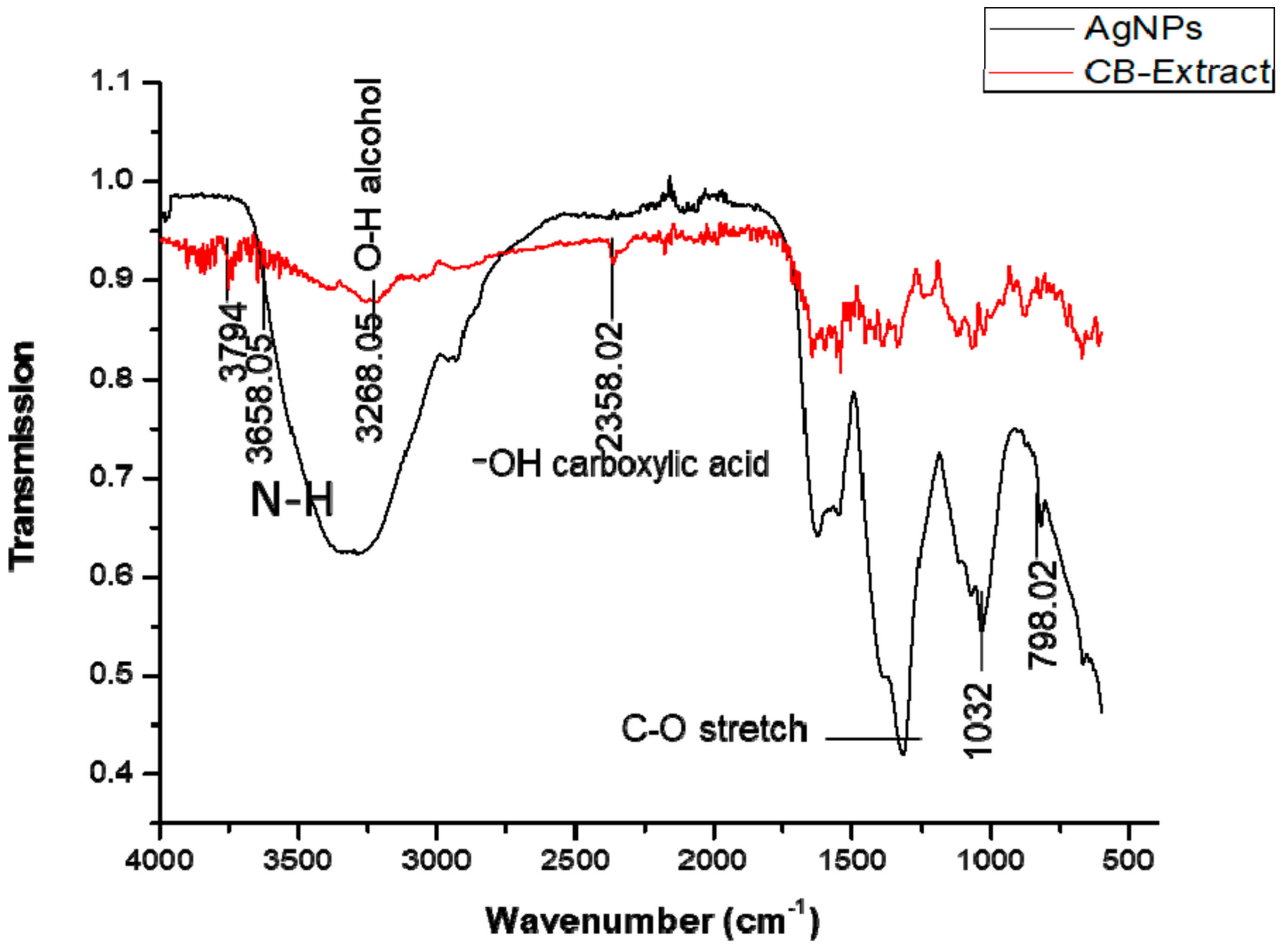
FTIR spectrum of biogenically synthesized silver nanoparticles with extract.

FTIR spectroscopy was used to analyze the surface and functional groups on phytochemicals and their interaction with the AgNPs. The FTIR analysis was carried out within a range of 400–4000 cm^−1^ (Figure 4). The FTIR spectra of *C. brychapara* had visible intensities at 3794.05, 3658, 3321, 2952, 1647, 1541, 1389, 1183, 1045, and 711 cm^−1^. Meanwhile, for Ag nanoparticles, the observable bands were at 3293, 2918, 1610, 1589, 1262, 1032, and 798.02 cm^−1^. The intense peak between 1800 and 1600 cm^−1^ indicated the presence of phenolics and the flavonoids group. The peaks shown at 1389 cm^−1^ correspond to C-N of the amide group, indicating the presence of proteins. The peak at 1189 cm^−1^ reduced and shifted toward 1032, which represents the phenol cluster. The observed peak at 798.02 described the formation of cation stabilization from oxygen. The strong infrared band (IR) near 3293 cm^−1^ indicated the hydroxyl group’s O–H vibration of phenols and flavonoids. In conclusion, the FTIR of the extract showed broader peaks that represent various -OH groups in flavonoids and phenolics. On that basis of above mentioned results we can conclude that Quercetin, Gallic acid, and flavone ascorbic acid could be involved in the bioreduction and capping agents for the synthesis of silver nanoparticles.

### 3.3. Scanning Electron Microscope

**Figure 5:**
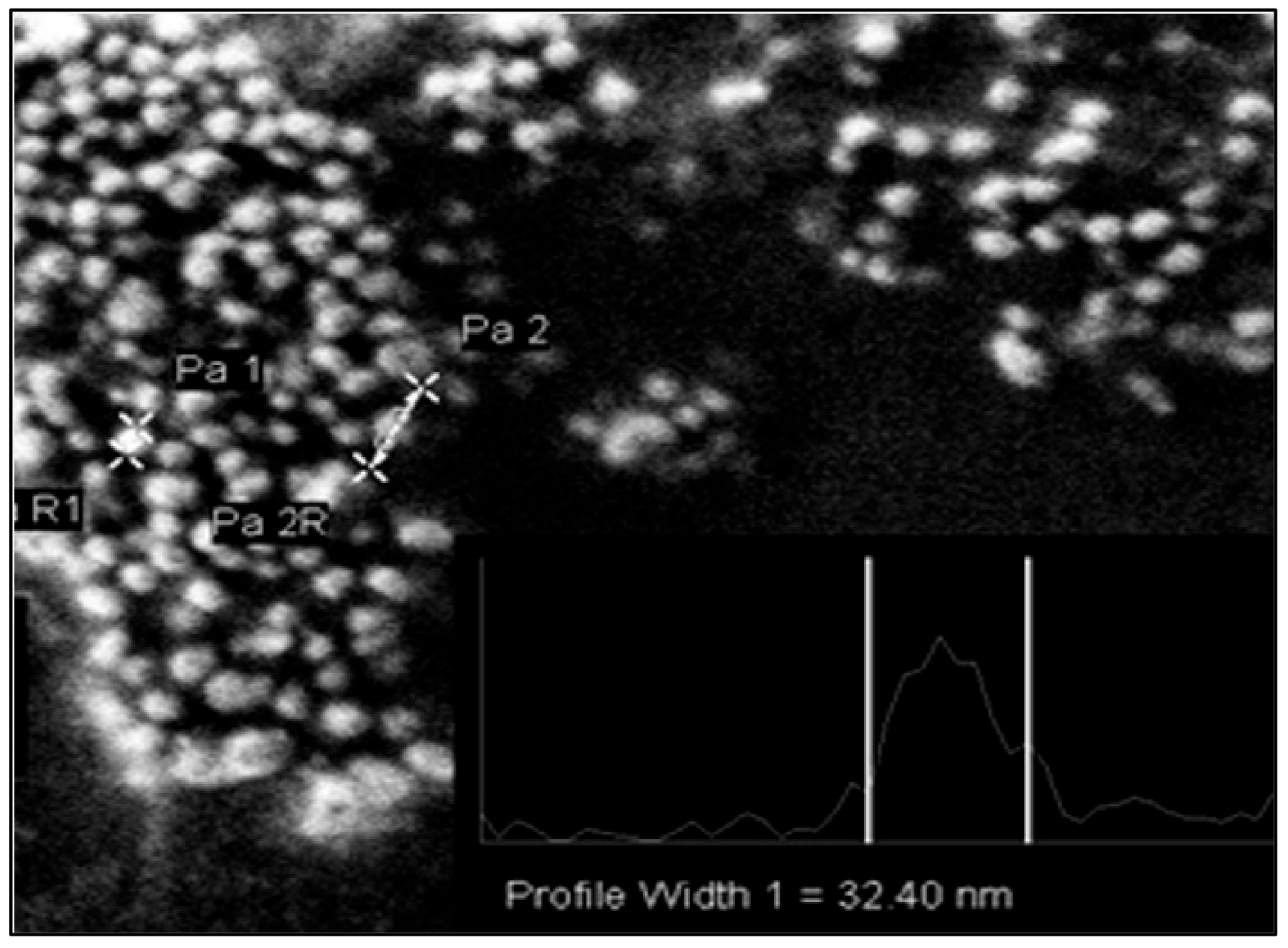
SEM image of CB-AgNPs obtained from *Cleome brachycarpa*.

The morphological appearance of biogenically synthesized AgNPs was determined through SEM analysis at 200 nm. It could be perceived that the median particle size of the AgNPs was mainly 20–80 nm, having spherical, uneven shapes due to slight agglomeration (Figure 5).

### 3.4. EDX Studies

**Figure 6:**
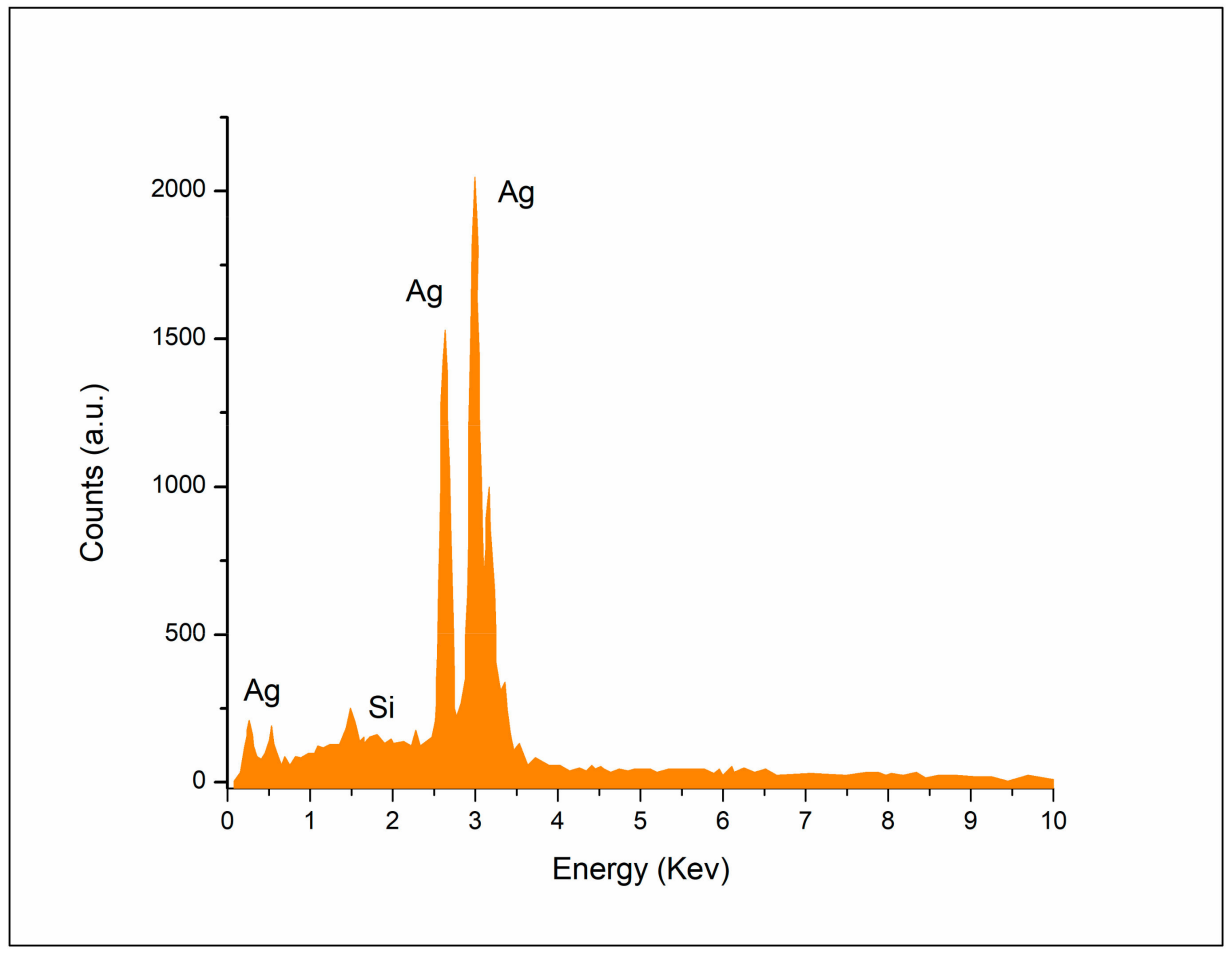
EDX spectrum of CB-AgNPs.

The elemental alignments of biogenically fabricated AgNPs were scanned with EDX, as depicted in Figure 6. The EDX analysis represented the presence of silver along with Si and a few other elements, which might be due to the presence of impurities introduced during the grinding process. The three strong peaks of Ag show the presence of silver at 0.8, 2.7, and 3 keV. These are typical absorption peaks of silver nanomaterials that corroborate with previous studies, as shown in Figure 6.

### 3.5. Atomic Force Microscopy (AFM) Analysis

**Figure 7:**
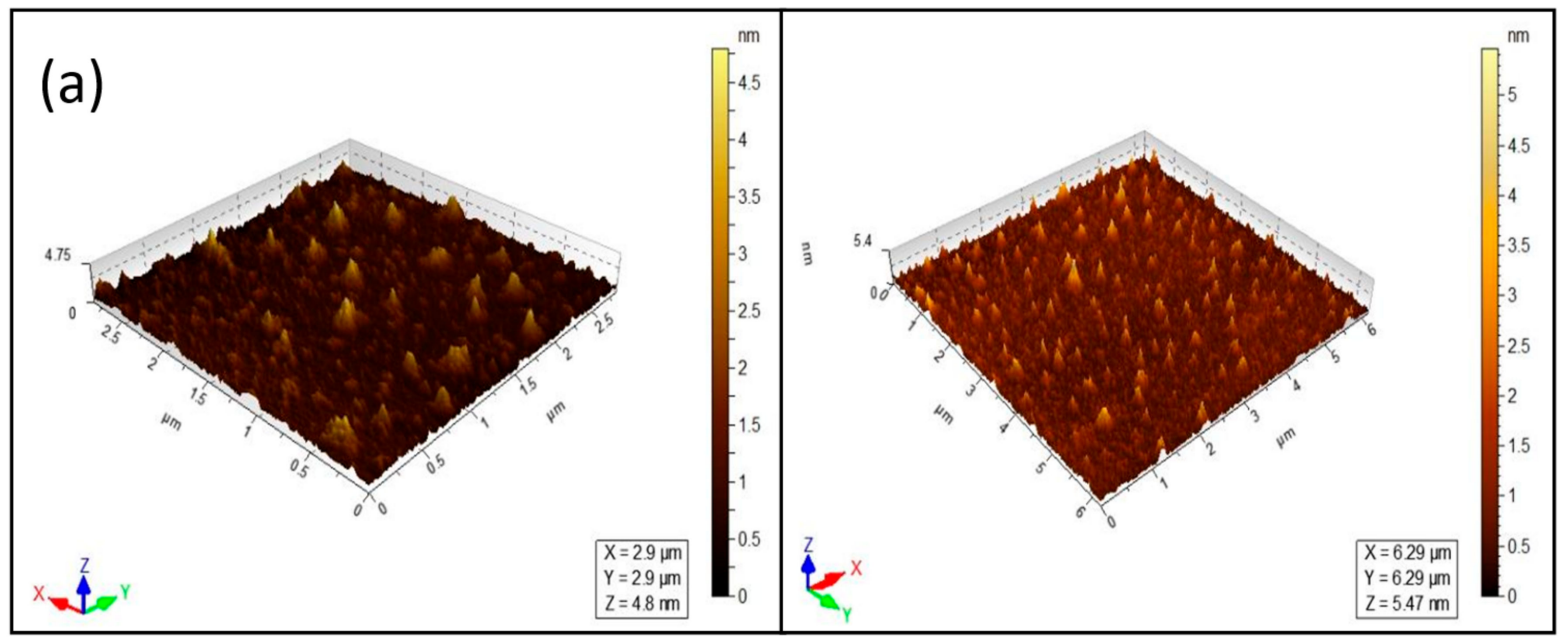

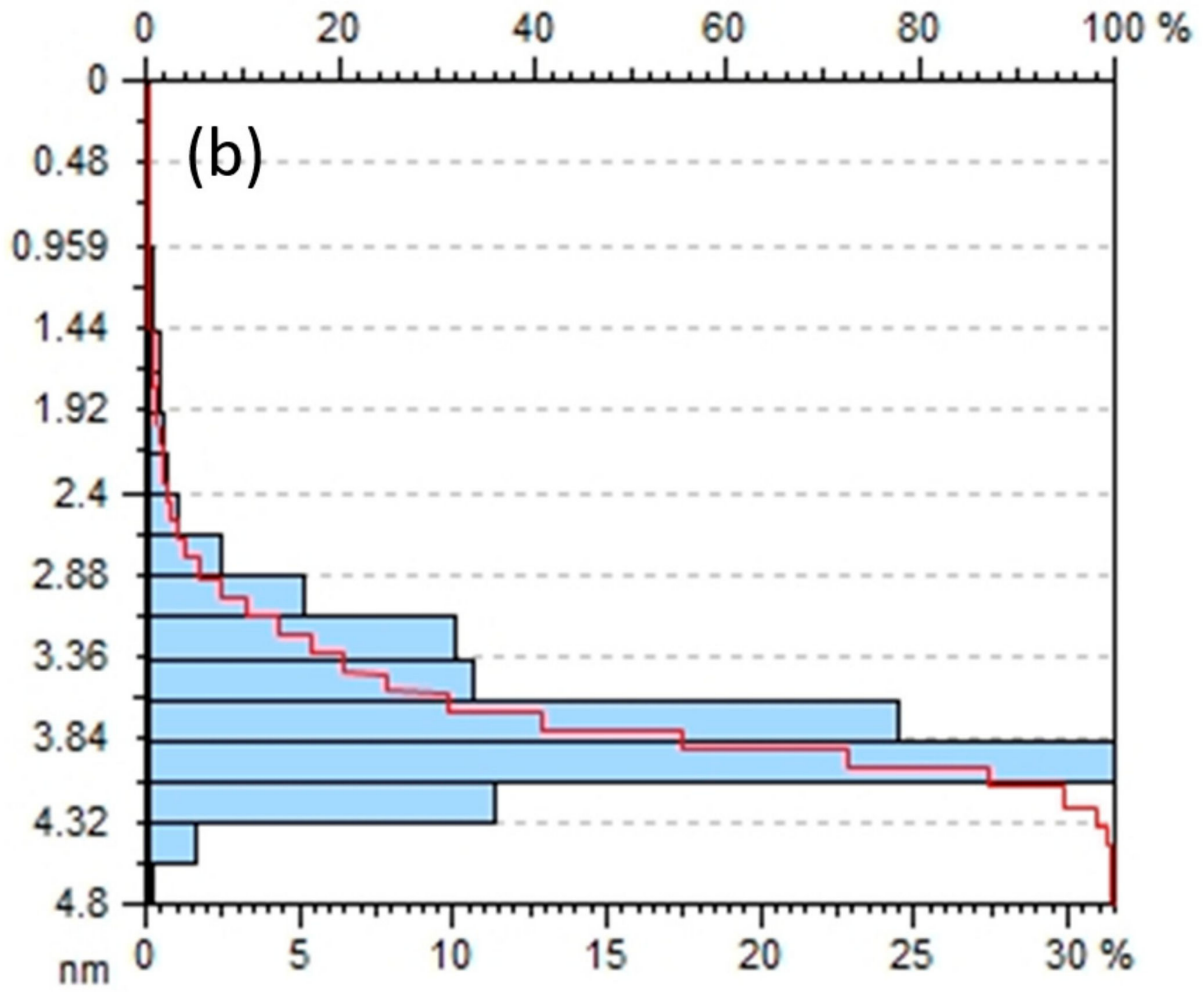
(**a**) 3D Atomic Force microscopy of silver nanoparticles; (**b**) average size range of AgNPs.

AFM analysis was employed to characterize the particle size and the morphology of AgNPs. Figure 7a shows the three-dimensional and topographical images of silver nanoparticles at two diverse nanometer ranges. Figure 7b shows the average size range of silver nanoparticles.

### 3.6. Antioxidant Activity

#### 3.6.1. The Antioxidant Activity DPPH and PFRAP

**Figure 8:**
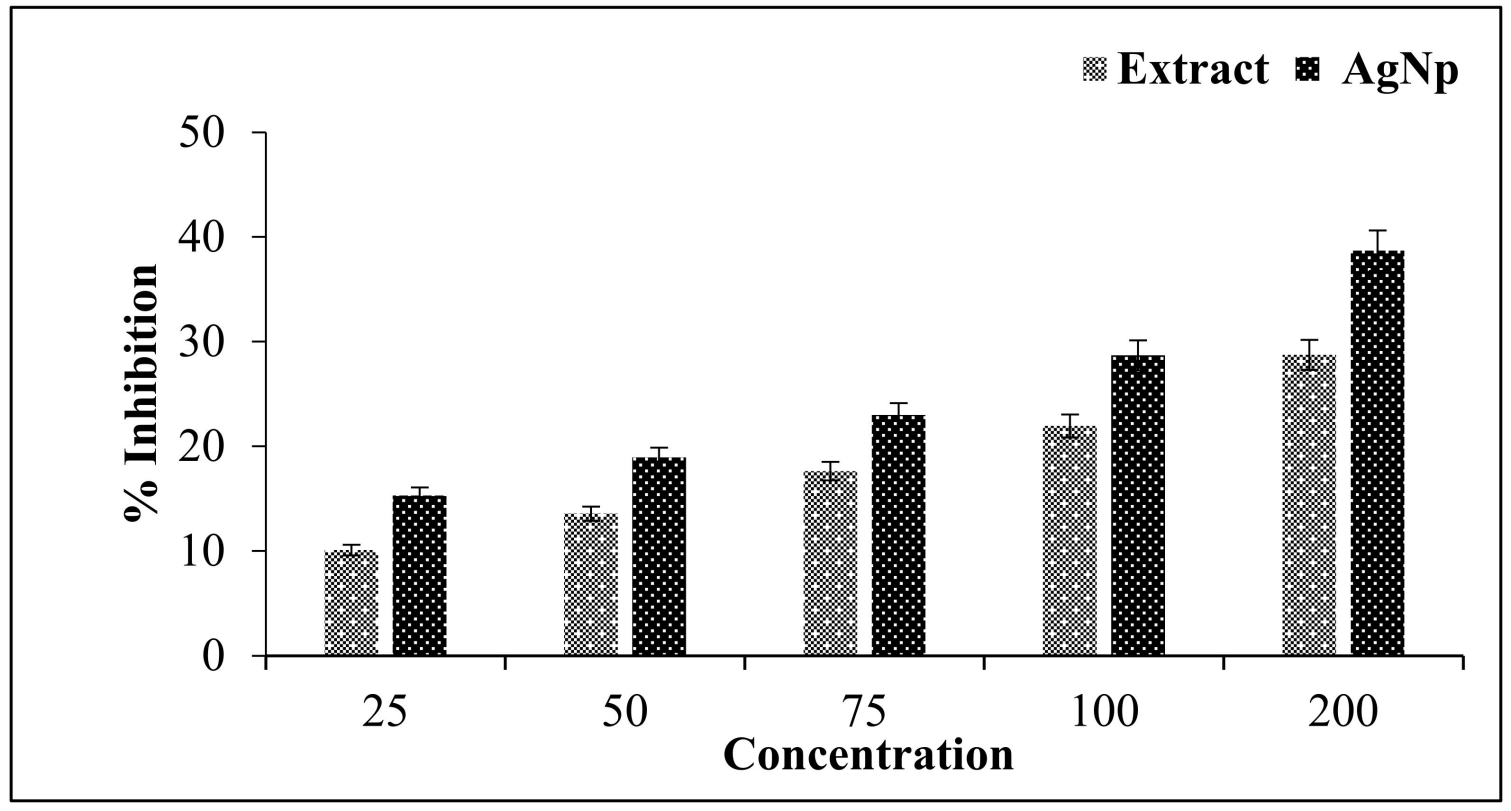
Antioxidant Potential of Extract with AgNPs.

**Figure 9:**
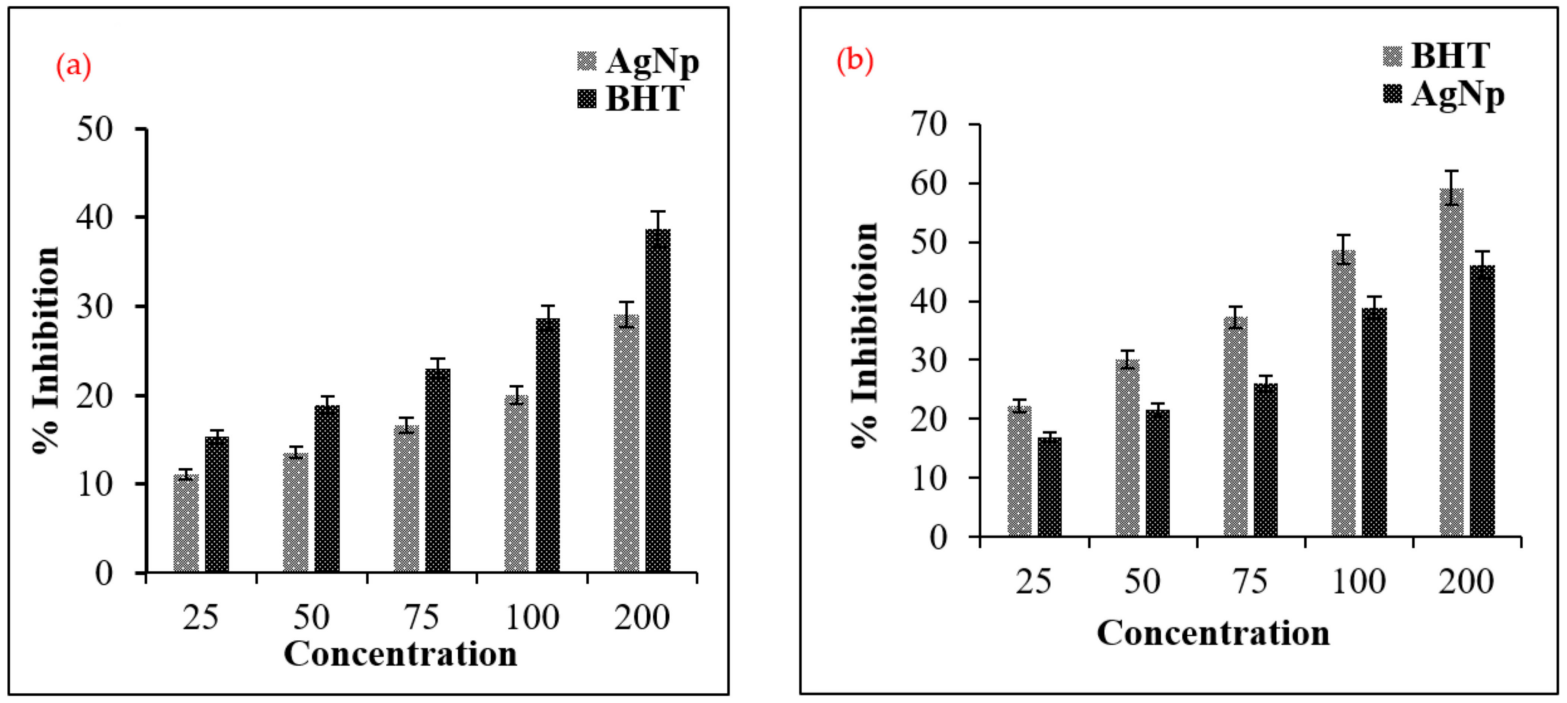
The activities of antioxidants using (**a**) DPPH (**b**) PFRAP assay.

By using DPPH and PFRAP assays, the scavenging potential of eco-friendly synthesized nanoparticles and *Cleome brachycarpa* extract were assessed, and the BHT was used as a standard. The outcomes from the two assays of antioxidant inhibition are compatible with one another. Our findings indicated that silver nanoparticles and *CB* extract have dose-dependent activity, as shown in Figure 8. These results show that AgNPs have a potential nearly equal to BHT as a standard at a concentration of 200 μg/mL, as determined through DPPH and PFRAP assays (Figure 9a,b).

For tested samples, the antioxidant potential results indicate the significant value of radical scavenging activity as RSA percent. The purpose of the antioxidant agent is to eliminate free radicals through its reduction by providing hydrogen atoms to free radicals that reduce DPPH radicals (purple color) into diphenylpicrylhydrazine (yellow color), which is the basis for detection using DPPH assay.

#### 3.6.2. Antidiabetic Activity

**Figure 10:**
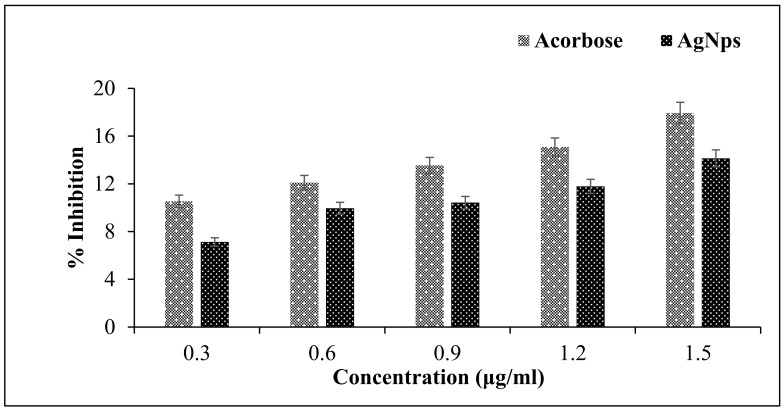
α-amylase activity of AgNPs.

AgNps from *C. brachycarpa* were also assessed for α-amylase activity as a measure of antidiabetic ability. In the stomach, carbohydrate digestive enzymes, i.e., pancreatic α-amylase, are liable for the breakdown of oligosaccharide into monosaccharides suitable for absorption. The outcomes revealed that AgNPs demonstrated the highest percentage value of inhibition on α-amylase activity. The graph in Figure 10 shows the percent inhibition of alpha-amylase according to different concentrations of AgNPs, indicating that α-amylase was significantly inhibited in a concentration-dependent manner.

#### 3.6.3. Cytotoxic Potential (MTT Assay)

**Figure 11:**
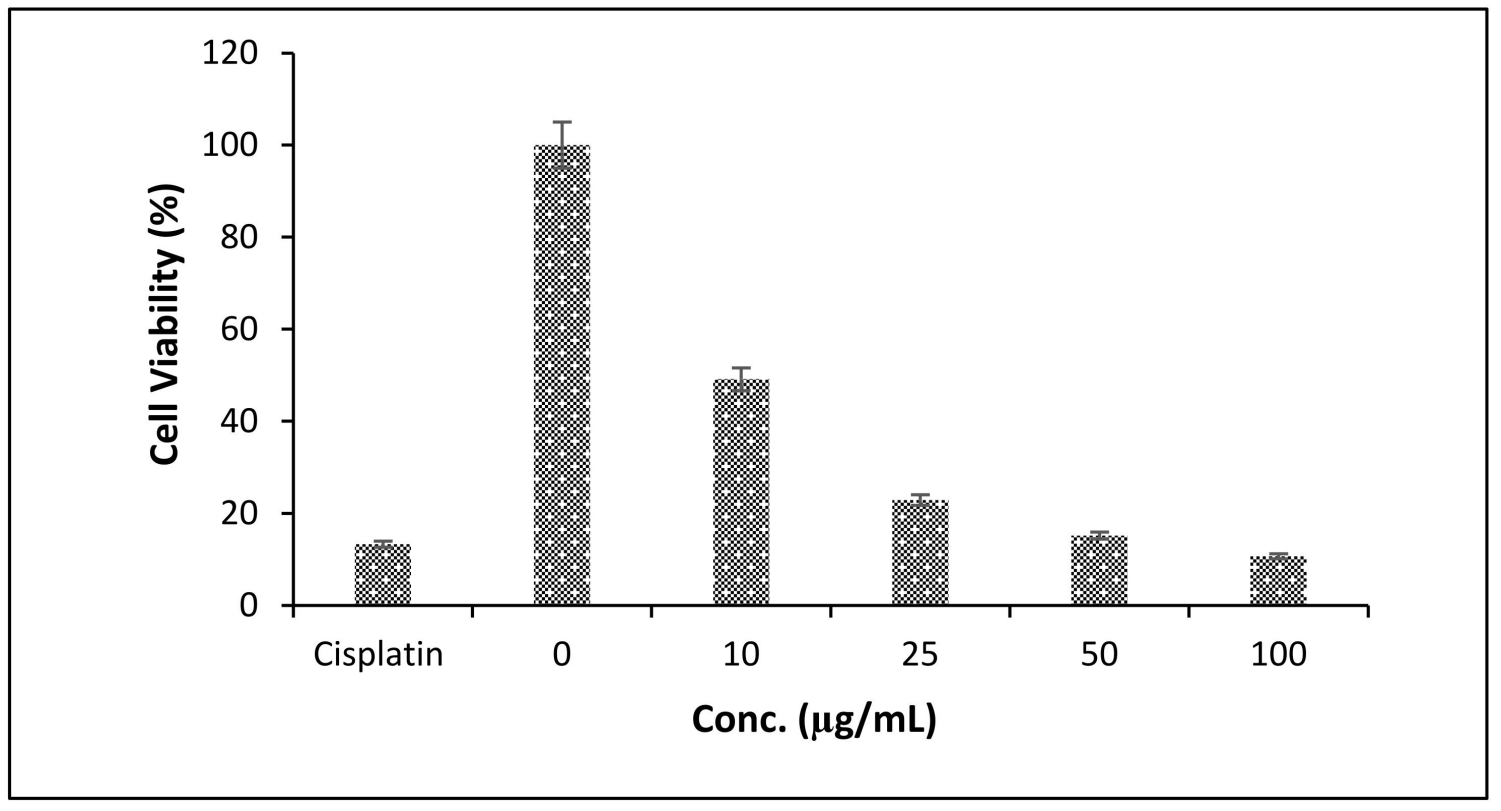
Anticancer potential of untreated control, cisplatin, and silver nanoparticles at different concentrations against MCF-7.

The AgNPs were evaluated for cytotoxic potential using MTT assay on the basis of the viability of cancer cells (breast cancer cell line (MCF-7)). Figure 11 shows MCF7 cells treated with different concentrations (0–100 μg/mL) of AgNPs and cisplatin for 24 h. Figure 11 shows that results from the MTT assay indicated that a significant reduction in viability was observed in MCF-7 following treatment with different concentrations (0–100 μg/mL) of silver nanoparticles with inhibition concentration (IC_50_) at 18 μg/mL, while cisplatin, a standard drug, showed a significant reduction of viability at 15 μg/mL.

## 4. Discussion

Phytochemicals are natural antioxidants derived from plants that can resist and protect themselves from free radicals and their reactive derivatives (ROS). The formation of biogenic AgNPs from plants has been reported by many researchers [6,41,42,43]. In the present work, AgNPs prepared using *C. brachycarpa* were screened for phytochemicals, including glycosides, alkaloids, flavonoids, proteins, tannins, phenols, terpenoids, steroids, and saponins because the presence of phytochemicals as secondary metabolites (phenolic, flavonoids, and terpenoids) play an important role in the pharmacological activities of plants [44]. Therefore, to produce new drugs to target human diseases, it is important to screen for phytochemical properties. The evidence for diverse phytoconstituents in *C. brachycarpa* with potential medicinal properties rationalizes our approach to developing novel *CB*-AgNPs in this study. The primary indication of synthesizing the nanoparticles is the change in color from yellow to dark brown (Figure 1) [33]. The highest absorption peak was observed at 437 nm, while the minimum peak was recorded at 190 nm, as demonstrated in Figure 2. This might be because one of the characteristics of surface plasmon resonance is associated with metal nanostructures. The SPR band caused by the excitation and AgNO_3_ is responsible for the color transformation of the reaction to reddish-brown. Optical bandgap value for synthesized AgNPs was calculated with a Tauc plot using absorption and wavelength and was converted into energy (Figure 3). The calculated Eg value for silver nanoparticles was 3.2 eV, which demonstrates that visible regions of the spectrum may be able to simulate electron transport in synthesized material [45]. The confirmation of the synthesis of *CB*-AgNPs from AgNO_3_ was performed via UV-visible spectral analysis of solution. In this study, to find the functional groups of molecules that are responsible for reducing silver ions and coating, reduced AgNP FTIR analysis was performed. Typically, the FTIR spectrum of synthesized *CB*-AgNPs shows numerous absorption bands, indicating different stretching and bending modes, including O-H, C-H, C=C, and C-O, as shown in Figure 4. The FTIR analysis shows that the *CB*-AgNPs are coated with phytochemicals from plant extract that contain a variety of organic molecule functional groups (flavonoids, phenolics, tannins, and terpenoids), as depicted in Figure 4, as reported earlier [46,47,48], because secondary metabolites and phytochemicals in high quantity also contribute to the pharmacological activities of these plants. As previously reported, C-H stretching vibration of the groups (CH_2_ and CH_3_) might prompt the absorption peak at 2918 cm^−1^ [49]. The peaks obtained at wavelengths of 1632 cm^−1^ and 1500 cm^−1^ indicate the formation of amide compounds. NH-stretching and peak intensity at a wavelength of 1032 cm^−1^ is attributed to C-O stretching from plant extracts, as supported by [50]. Scanning electron microscopy revealed the size and morphology of the nanoparticles. The size of *CB*-AgNPs was 20–80 nm and they were spherical in shape, which might be due to slight agglomeration in the plant extract (Figure 5). Moreover, carbon and oxygen were also detected. EDX analysis showed the maximum percent of Ag (Figure 6), because *CB* extract (which is specifically rich in flavonoids & phenolic entities) acts as a reducing agent in an artificial process, and this result is in agreement with previous findings [29]. AFM analysis showed the surface area and size distribution of silver nanoparticles, as depicted in Figure 7a,b [51].

The antioxidant properties of purified *CB*-AgNPs were determined in vitro through analysis reducing power assay, and by measuring DPPH scavenging activity. Figure 8 shows that both *CB*-AgNPs and extracts were very important in diminishing the scavenging and reduction of free radicals. Similarly, *CB*-AgNPs with different concentrations compared to a standard showed effective activity of DPPH scavenging. DPPH scavenging assay is an effective method to determine the antioxidant activity (in vitro). DPPH is also used to assess the scavenging capacity following the mechanism that blocks the oxidation of lipids. In addition, it is a stable substance that receives hydrogen from silver nanostructures [52]. Thus, the radical compound is stable and need not be generated. There is a quick response of DPPH with phenols. The conversion of the purple color of the DPPH solution into yellow is due to the allocation of an electron to DPPH radicals from an antioxidant caused by unpaired electrons present in the DPPH solution. This change in coloration was determined via spectrophotometric analysis [53]. *CB*-AgNPs showed momentous DPPH scavenging activity compared to *CB*-extract (Figure 8) because of their high secondary metabolites (flavonoids, phenolic, terpenoids, and alkaloids) [29]. It was observed that with the increase in concentration, the antioxidant activity of *CB*-AgNPs increased (Figure 9a), which was comparable with and in agreement with previous findings [54,55].

A reducing power assay was also used to measure antioxidant potential, in which the reduction of Fe^+3^ with a yellow color to Fe^2+^ with a blue color by transfer of an electron instead of a hydrogen atom was observed. The reducing potential also confirmed the antioxidant activity of nanoparticles as compared to extract. The Fe^+3^ reduced to Fe^+2^ with an absorption maximum at 593 nm. For determination of the reducing power, *CB*-AgNPs and BHT were also examined, as shown in Figure 9b. The *CB*-extract showed a lower reducing ability than *CB*-AgNPs. Both *CB*-extract and *CB*-AgNPs showed a concentration-dependent reducing capacity, as described in previous studies [56]. In the same way, AgNPs were also used to demonstrate in vitro antidiabetic activity, as shown in Figure 10. Diabetes is a category of metabolic disorders characterized by persistently elevated blood glucose levels, which can result in many diseases [57]. Theranostic methods help to decrease hyperglycemia by inhibiting the activity of α-amylase (a carbohydrate-digesting enzyme that increases breakdown into mono-saccharides and contributes to increased blood glucose levels) [57,58]. Acarbose is a glucosidase inhibitor that inhibits human pancreatic amylase. With a delay in carbohydrate digestion, acarbose shows low glucose absorption resulting from the reduction of glucose concentration in blood [59].

Our results show the antidiabetic ability of AgNPs with effective inhibition at low concentrations against carbohydrate digestive enzymes such as α-amylase. This may be a result of the presence of potential α-amylase inhibitors. Previous findings showing the antidiabetic ability of AgNPs with α-glucosidase/amylase inhibition support our results [60,61,62]. In summary, although generally lower compared to corresponding precursors, this study revealed that AgNPs have interesting enzymatic activity, and showed that their significant inhibitory activities are concentration-dependent.

The most significant requirements of cancer therapeutics are selective cytotoxicity and efficacy. A medicine’s ability to cause the apoptosis of cancer cells makes it a successful cancer treatment. Candidate drugs should not negatively influence immune system cells, as these agents attack cancer in conjunction with the immune system. To better understand these effects, silver nanoparticles were tested in MCF-7 cells (human breast cancer cells).The MTT results showed the cytotoxic potential of AgNPs using a colorimetric assay to assess for color changes (purple to yellow) upon reduction of formazan in the presence of mitochondrial succinate dehydrogenase (Figure 11). Similarly, previous findings supported this study regarding the inhibition concentration of AgNPs being 18 μg/mL [40]. In agreement with previous reports, at lower concentrations, toxicity is negligible [47,63,64]. However, the foregoing results suggest that the synthesized AgNPs could be potentially useful in synthesizing diabetes-related drugs, as well as could be a potential substitute for human breast cancer therapy.

## 5. Conclusions

In short, the biologically active silver nanoparticles were produced via green synthesis in an effective and supportive technique using *Cleome brachycarpa* extract for the first time. Due to the presence of dynamic phytoconstituents, the nanoparticles provided additional benefits. The synthesized silver nanoparticles were confirmed via different studies. UV-visible spectroscopy showed a maximum absorption peak at 437 nm, which confirms the presence of AgNPs. FTIR showed different functional peaks present in the extract responsible for the reduction and induction of metal–oxygen bonds shown in organic regions. SEM showed the morphology of silver nanoparticles along with elemental composition, as confirmed by EDX. The *CB*-AgNPs were determined to be valid based on their valuable potential effects on free radicals. Capped phytochemical compounds were responsible for the antioxidant properties of *CB*-AgNPs, showing the broad potential of silver nanoparticles. Therefore, using *CB*-AgNPs for a series of applications as a remedial agent to combat free radical-mediated losses shows promise. Furthermore, this study revealed that these results are associated with considerable amounts of phytochemicals such as flavonoids, saponins, tannins, and polyphenolic compounds in *CB* extract. *C. brachycarpa* extract-mediated silver nanoparticles may be useful for other biomedical applications, as they showed outstanding efficiency for inhibitory action, scavenging potential, and strong anticancer activity against MCF-7, potentially making them therapeutic alternative agents for human breast cancer treatment. In the future of nanomedicine, AgNPs designed from therapeutic plant extract may be implemented as a medical device.

## Figures and Tables

**Table 1:** Phytochemical screening tests and their indications.

Sr no.	Phytochemicals	Screening Test	Indication
1	Alkaloids	0.5 mL extract +2 mL HCl + 1 mL Dragendroff’s reagent	Orange or red precipitates
2	Saponins	0.5 mL extract + 2 mL distilled H_2_O + shaken vigorously	Appearance of foam layer
3	Phenols	0.5 mL extract + 5 mL distilled H_2_O + 2–3 drops of neutral 5% FeCl_3_ solution	Bluish-black color
4	Flavonoids	1 mL extract + 1 mL 10% Pb(C_2_H_3_O_2_)_2_ solution	Yellow precipitates
5	Tannins	0.5 mL extract + 1 mL 10% K_2_Cr_2_O_7_ solution	Yellow precipitates
6	Proteins	1 mL extract + 2 drops Ninhydrin’s reagent	Blue color
7	Steroids	1 mL extract + 10 mL CHCl_3_ + 10 mL conc. H_2_SO_4_ sides of test tube	Upper layer red and H_2_SO_4_ layer green fluorescence
8	Terpenoids	2 mL extract + 2 mL CHCl_3_ + 3 mL conc. H_2_SO_4_ + 2 min heating	Reddish-brown color
9	Glycosides	0.5 mL extract + 2 mL glacial CH_3_COOH + 1–2 drops 2% FeCl_3_ + 2 mL conc. H_2_SO_4_	Brown ring at junction

**Table 2:** Phytochemicals present in *Cleome brachycarpa* aqueous extract.

Sr.no	Phytochemicals	Presence (+)/Absence (-)
01	Phenolics	+
02	Flavonoids	+
03	Saponins	+
04	Alkaloids	+
05	Tannins	+
06	Steroids	+
07	Glycosides	-
08	Terpenoids	+
09	Proteins	+

## Data Availability

All data supporting this research article are already included in this manuscript.

## Supplementary Materials

The following supporting information can be downloaded at: https://www.mdpi.com/article/10.3390/plants12071578/s1, Supplementary Data.

## References

[1] Ahmad S., Munir S., Zeb N., Ullah A., Khan B., Ali J., Bilal M., Omer M., Alamzeb M., Salman S.M. (2019). Green nanotechnology: A review on green synthesis of silver nanoparticles—An ecofriendly approach. Int. J. Nanomed..

[2] Babayevska N., Przysiecka Ł., Iatsunskyi I., Nowaczyk G., Jarek M., Janiszewska E., Jurga S. (2022). ZnO size and shape effect on antibacterial activity and cytotoxicity profile. Sci. Rep..

[3] Lakshmanan G., Sathiyaseelan A., Kalaichelvan P.T., Murugesan K. (2018). Plant-mediated synthesis of silver nanoparticles using fruit extract of *Cleome viscosa* L.: Assessment of their antibacterial and anticancer activity. Karbala Int. J. Mod. Sci..

[4] Nguyen T.H.A., Nguyen V.-C., Phan T.N.H., Le V.T., Vasseghian Y., Trubitsyn M.A., Nguyen A.-T., Chau T.P., Doan V.-D. (2021). Novel biogenic silver and gold nanoparticles for multifunctional applications: Green synthesis, catalytic and antibacterial activity, and colorimetric detection of Fe(III) ions. Chemosphere.

[5] Vijayaraghavan K., Ashokkumar T. (2017). Plant-mediated biosynthesis of metallic nanoparticles: A review of literature, factors affecting synthesis, characterization techniques and applications. J. Environ. Chem. Eng..

[6] Shah M., Nawaz S., Jan H., Uddin N., Ali A., Anjum S., Giglioli-Guivarc’h N., Hano C., Abbasi B.H. (2020). Synthesis of bio-mediated silver nanoparticles from *Silybum marianum* and their biological and clinical activities. Mater. Sci. Eng. C.

[7] Mobaraki F., Momeni M., Jahromi M., Kasmaie F.M., Barghbani M., Yazdi M.E.T., Meshkat Z., Shandiz F.H., Hosseini S.M. (2022). Apoptotic, antioxidant and cytotoxic properties of synthesized AgNPs using green tea against human testicular embryonic cancer stem cells. Process Biochem..

[8] Busatto S., Pham A., Suh A., Shapiro S., Wolfram J. (2019). Organotropic drug delivery: Synthetic nanoparticles and extracellular vesicles. Biomed. Microdevices.

[9] Ansari M.A., Yadav M.K., Rathore D., Svedberg A., Karim Z. (2019). Applications of Nanostructured Polymer Composites for Gene Delivery. Nanostructured Polymer Composites for Biomedical Applications.

[10] Dikshit P., Kumar J., Das A., Sadhu S., Sharma S., Singh S., Gupta P., Kim B. (2021). Green Synthesis of Metallic Nanoparticles: Applications and Limitations. Catalysts.

[11] Shumail H., Khalid S., Ahmad I., Khan H., Amin S., Ullah B. (2021). Review on Green Synthesis of Silver Nanoparticles through Plants. Endocr. Metab. Immune Disord.—Drug Targets.

[12] Ali Z.A., Yahya R., Sekaran S.D., Puteh R. (2016). Green Synthesis of Silver Nanoparticles Using Apple Extract and Its Antibacterial Properties. Adv. Mater. Sci. Eng..

[13] Baharara J., Namvar F., Ramezani T., Mousavi M., Mohamad R. (2015). Silver Nanoparticles Biosynthesized Using *Achillea biebersteinii* Flower Extract: Apoptosis Induction in MCF-7 Cells via Caspase Activation and Regulation of Bax and Bcl-2 Gene Expression. Molecules.

[14] Salehi S., Shandiz S.A., Ghanbar F., Darvish M.R., Ardestani M.S., Mirzaie A., Jafari M. (2016). Phytosynthesis of silver nanoparticles using *Artemisia marschalliana* Sprengel aerial part extract and assessment of their antioxidant, anticancer, and antibacterial properties. Int. J. Nanomed..

[15] Aktepe N., Baran A. (2021). Fast and low-cost biosynthesis of AgNPs with almond leaves: Medical applications with biocompatible structures. Prog. Nutr..

[16] Yazdi M.E.T., Amiri M.S., Akbari S., Sharifalhoseini M., Nourbakhsh F., Mashreghi M., Yousefi E., Abbasi M.R., Modarres M., Es-Haghi A. (2020). Green Synthesis of Silver Nanoparticles Using *Helichrysum graveolens* for Biomedical Applications and Wastewater Treatment. Bionanoscience.

[17] Anbarasu A., Karnan P., Deepa N., Usha R. (2018). Carica papaya mediated green synthesized silver nanoparticles. Int. J. Curr. Pharm. Res..

[18] Moteriya P., Chanda S. (2020). Green Synthesis of Silver Nanoparticles from *Caesalpinia pulcherrima* Leaf Extract and Evaluation of Their Antimicrobial, Cytotoxic and Genotoxic Potential (3-in-1 System). J. Inorg. Organomet. Polym. Mater..

[19] Yarrappagaari S., Gutha R., Narayanaswamy L., Thopireddy L., Benne L., Mohiyuddin S.S., Vijayakumar V., Saddala R.R. (2020). Eco-friendly synthesis of silver nanoparticles from the whole plant of *Cleome viscosa* and evaluation of their characterization, antibacterial, antioxidant and antidiabetic properties. Saudi J. Biol. Sci..

[20] Rai M., Ingle A.P., Gade A., Duran N. (2015). Synthesis of silver nanoparticles by *Phoma gardeniae* and in vitro evaluation of their efficacy against human disease-causing bacteria and fungi. IET Nanobiotechnology.

[21] Ahmed R.H., Mustafa D.E. (2020). Green synthesis of silver nanoparticles mediated by traditionally used medicinal plants in Sudan. Int. Nano Lett..

[22] Ahmad V.U., Qazi S., Bin Zia N., Xu C., Clardy J. (1990). Cleocarpone, a triterpenoid from *Cleome brachycarpa*. Phytochemistry.

[23] Rassouli E., Dadras O.G., Bina E., Asgarpanah J. (2014). The Essential Oil Composition of *Cleome brachycarpa* Vahl ex DC. J. Essent. Oil Bear. Plants.

[24] Hameed M., Ashraf M., Al-Quriany F., Nawaz T., Ahmad M.S.A., Younis A., Naz N. (2011). Medicinal flora of the Cholistan desert: A review. Pak. J. Bot..

[25] Chand J., Panda S.R., Jain S., Murty U., Das A.M., Kumar G.J., Naidu V. (2021). Phytochemistry and polypharmacology of cleome species: A comprehensive Ethnopharmacological review of the medicinal plants. J. Ethnopharmacol..

[26] Facciola S. (1990). Cornucopia: A Source Book of Edible Plants.

[27] Nguyen T.P., Tran C.L., Vuong C.H., Do T.H.T., Le T.D., Mai D.T., Phan N.M. (2017). Flavonoids with hepatoprotective activity from the leaves of *Cleome viscosa* L. Nat. Prod. Res..

[28] Upadhyay R. (2015). *Cleome viscosa* Linn: A natural source of pharmaceuticals and pesticides. Int. J. Green Pharm..

[29] Saleem T., Sumra A., Khan S., Zain M., Hassan W., Mehdi S., Wahid N.-U., Kanwal S., Gull T. (2020). A Green Nutraceutical Study of Antioxidants Extraction in *Cleome brachycarpa*—An Ethnomedicinal Plant. Sains Malays..

[30] Benakashani F., Allafchian A., Jalali S. (2016). Biosynthesis of silver nanoparticles using *Capparis spinosa* L. leaf extract and their antibacterial activity. Karbala Int. J. Mod. Sci..

[31] World Health Organization (1998). Quality Control Methods for Medicinal Plant Materials.

[32] Al-Rajhi A.M., Salem S.S., Alharbi A.A., Abdelghany T. (2022). Ecofriendly synthesis of silver nanoparticles using Kei-apple (*Dovyalis caffra*) fruit and their efficacy against cancer cells and clinical pathogenic microorganisms. Arab. J. Chem..

[33] Das G., Patra J.K., Debnath T., Ansari A., Shin H.-S. (2019). Investigation of antioxidant, antibacterial, antidiabetic, and cytotoxicity potential of silver nanoparticles synthesized using the outer peel extract of *Ananas comosus* (L.). PLoS ONE.

[34] Patra J.K., Das G., Kumar A., Ansari A., Kim H., Shin H.-S. (2018). Photo-mediated Biosynthesis of Silver Nanoparticles Using the Non-edible Accrescent Fruiting Calyx of *Physalis peruviana* L. Fruits and Investigation of its Radical Scavenging Potential and Cytotoxicity Activities. J. Photochem. Photobiol. B Biol..

[35] Pyrzynska K., Pękal A. (2013). Application of free radical diphenylpicrylhydrazyl (DPPH) to estimate the antioxidant capacity of food samples. Anal. Methods.

[36] Layer P., Rizza R.A., Zinsmeister A.R., Carlson G.L., DiMAGNO E.P. (1986). Effect of a Purified Amylase Inhibitor on Carbohydrate Tolerance in Normal Subjects and Patients with Diabetes Mellitus. Mayo Clin. Proc..

[37] Khashan K.S., Sulaiman G.M., Hussain S.A., Marzoog T.R., Jabir M.S. (2020). Synthesis, Characterization and Evaluation of Anti-bacterial, Anti-parasitic and Anti-cancer Activities of Aluminum-Doped Zinc Oxide Nanoparticles. J. Inorg. Organomet. Polym. Mater..

[38] Al-Salman H.N.K., Ali E., Jabir M., Sulaiman G.M., Al-Jadaan S.A.S. (2020). 2-Benzhydrylsulfinyl-N-hydroxyacetamide-Na extracted from fig as a novel cytotoxic and apoptosis inducer in SKOV-3 and AMJ-13 cell lines via P53 and caspase-8 pathway. Eur. Food Res. Technol..

[39] Maskell L., Mahadeo A., Budhram-Mahadeo V. (2018). POU4F2/Brn-3b transcription factor is associated with survival and drug resistance in human ovarian cancer cells. Oncotarget.

[40] Gomathi A., Rajarathinam S.X., Sadiq A.M., Rajeshkumar S. (2019). Anticancer activity of silver nanoparticles synthesized using aqueous fruit shell extract of *Tamarindus indica* on MCF-7 human breast cancer cell line. J. Drug Deliv. Sci. Technol..

[41] Zhang Y.-J., Gan R.-Y., Li S., Zhou Y., Li A.-N., Xu D.-P., Li H.-B. (2015). Antioxidant Phytochemicals for the Prevention and Treatment of Chronic Diseases. Molecules.

[42] Rashmi B., Harlapur S.F., Avinash B., Ravikumar C., Nagaswarupa H., Kumar M.A., Gurushantha K., Santosh M. (2019). Facile green synthesis of silver oxide nanoparticles and their electrochemical, photocatalytic and biological studies. Inorg. Chem. Commun..

[43] Dhar S.A., Alam Chowdhury R., Das S., Nahian K., Islam D., Gafur A. (2021). Plant-mediated green synthesis and characterization of silver nanoparticles using *Phyllanthus emblica* fruit extract. Mater. Today Proc..

[44] Saratale R.G., Saratale G.D., Shin H.S., Jacob J.M., Pugazhendhi A., Bhaisare M., Kumar G. (2017). New insights on the green synthesis of metallic nanoparticles using plant and waste biomaterials: Current knowledge, their agricultural and environmental applications. Environ. Sci. Pollut. Res..

[45] Sumra A.A., Aadil M., Ejaz S.R., Anjum S., Saleem T., Zain M., Alsafari I.A. (2022). Biological synthesis of nanostructured ZnO as a solar-light driven photocatalyst and antimicrobial agent. Ceram. Int..

[46] Qais F.A., Shafiq A., Khan H.M., Husain F.M., Khan R.A., Alenazi B., Alsalme A., Ahmad I. (2019). Antibacterial Effect of Silver Nanoparticles Synthesized Using *Murraya koenigii* (L.) against Multidrug-Resistant Pathogens. Bioinorg. Chem. Appl..

[47] Sabapathi N., Ramalingam S., Aruljothi K.N., Lee J., Barathi S. (2023). Characterization and Therapeutic Applications of Biosynthesized Silver Nanoparticles Using *Cassia auriculate* Flower Extract. Plants.

[48] Ahmad A., Javed M.S., Khan S., Almutairi T.M., Mohammed A.A., Luque R. (2022). Green synthesized Ag decorated CeO_2_ nanoparticles: Efficient photocatalysts and potential antibacterial agents. Chemosphere.

[49] Alsafari I.A., Chaudhary K., Warsi M.F., Warsi A.Z., Waqas M., Hasan M., Jamil A., Shahid M. (2023). A facile strategy to fabricate ternary WO3/CuO/rGO nano-composite for the enhanced photocatalytic degradation of multiple organic pollutants and antimicrobial activity. J. Alloys Compd..

[50] Anwar M., Alghamdi K.S., Zulfiqar S., Warsi M.F., Waqas M., Hasan M. (2023). Ag-decorated BiOCl anchored onto the g-C_3_N_4_ sheets for boosted photocatalytic and antimicrobial activities. Opt. Mater..

[51] Jain N., Jain P., Rajput D., Patil U.K. (2021). Green synthesized plant-based silver nanoparticles: Therapeutic prospective for anticancer and antiviral activity. Micro Nano Syst. Lett..

[52] Keerthiga N., Anitha R., Rajeshkumar S., Lakshmi T. (2019). Antioxidant Activity of Cumin Oil Mediated Silver Nanoparticles. Pharmacogn. J..

[53] Phaniendra A., Jestadi D.B., Periyasamy L. (2015). Free Radicals: Properties, Sources, Targets, and Their Implication in Various Diseases. Indian J. Clin. Biochem..

[54] Das G., Shin H.-S., Patra J.K. (2022). Comparative Bio-Potential Effects of Fresh and Boiled Mountain Vegetable (Fern) Extract Mediated Silver Nanoparticles. Plants.

[55] Saravanakumar A., Ganesh M., Jayaprakash J., Jang H.T. (2015). Biosynthesis of silver nanoparticles using *Cassia tora* leaf extract and its antioxidant and antibacterial activities. J. Ind. Eng. Chem..

[56] Reddy N.J., Vali D.N., Rani M., Rani S.S. (2014). Evaluation of antioxidant, antibacterial and cytotoxic effects of green synthesized silver nanoparticles by Piper longum fruit. Mater. Sci. Eng. C.

[57] Saratale R.G., Shin H.S., Kumar G., Benelli G., Kim D.-S., Saratale G.D. (2017). Exploiting antidiabetic activity of silver nanoparticles synthesized using *Punica granatum* leaves and anticancer potential against human liver cancer cells (HepG2). Artif. Cells Nanomed. Biotechnol..

[58] Etxeberria U., de la Garza A.L., Campión J., Martínez J.A., Milagro F.I. (2012). Antidiabetic effects of natural plant extracts via inhibition of carbohydrate hydrolysis enzymes with emphasis on pancreatic alpha amylase. Expert Opin. Ther. Targets.

[59] Wettergreen S.A., Sheth S., Malveaux J. (2016). Effects of the addition of acarbose to insulin and non-insulin regimens in veterans with type 2 diabetes mellitus. Pharm. Pract..

[60] Jini D., Sharmila S. (2020). Green synthesis of silver nanoparticles from *Allium cepa* and its in vitro antidiabetic activity. Mater. Today Proc..

[61] Patra J.K., Das G., Shin H.S. (2019). Facile green biosynthesis of silver nanoparticles using *Pisum sativum* L. outer peel aqueous extract and its antidiabetic, cytotoxicity, antioxidant, and antibacterial activity. Int. J. Nanomed..

[62] Rani R., Sharma D., Chaturvedi M., Yadav J.P. (2017). Green Synthesis, Characterization and Antibacterial Activity of Silver Nanoparticles of Endophytic Fungi *Aspergillus terreus*. J. Nanomed. Nanotechnol..

[63] Mani M., Okla M.K., Selvaraj S., Kumar A.R., Kumaresan S., Muthukumaran A., Kaviyarasu K., El-Tayeb M.A., Elbadawi Y.B., Almaary K.S. (2021). A novel biogenic Allium cepa leaf mediated silver nanoparticles for antimicrobial, antioxidant, and anticancer effects on MCF-7 cell line. Environ. Res..

[64] Rashidipour M., Heydari R. (2014). Biosynthesis of silver nanoparticles using extract of olive leaf: Synthesis and in vitro cytotoxic effect on MCF-7 cells. J. Nanostructure Chem..

