# Supplementary material for: Biogenic Synthesis, Characterization, and In Vitro Biological Evaluation of Silver Nanoparticles Using Cleome brachycarpa
Source: Plants (Basel). 2023 Apr 6;12(7):1578. doi: 10.3390/plants12071578 (PMC10097212; doi:10.3390/plants12071578)

University of Science & Technology, Bannu

Main Campus, Township Bannu, Ph # 0928-633321, www.ustb.edu.pk

Department of Botany

IDENTIFICATION CERTIFICATE

Certified that following plants are identified and verified by the undersign based on their vegetative ad floral characters.

1. *Cleome viscosa* Voucher No# Cv-C1
2. *Cleome brychycarpa* Voucher No# Cb-C2

18/9/19.

Prof.Dr.Sultan Mehmood
Department of Botany
UST Bannu

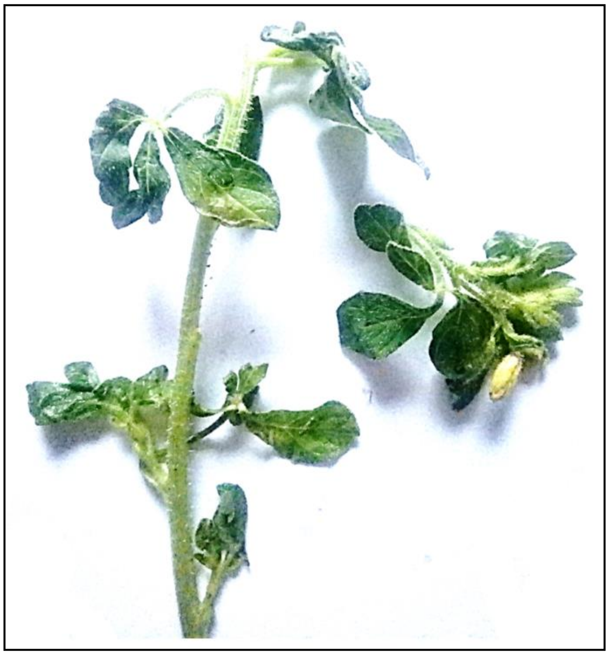

Supplement: Supplementary file 1 [file plants-12-01578-s001.zip › plants-2191320-Supplementary Data.pdf]
